# Role of Nrf2/HO-1 and cytoglobin signaling in the protective effect of indole-3-acetic acid and chenodeoxycholic acid against kidney injury induced by valproate

**DOI:** 10.1016/j.heliyon.2024.e41069

**Published:** 2024-12-07

**Authors:** Ahlam M. Alhusaini, Wedad Sarawi, Noor Mukhtar, Danah Aljubeiri, Amjad S. Aljarboa, Hessa Alduhailan, Faris Almutairi, Raeesa Mohammad, Muhammad Atteya, Iman Hasan

**Affiliations:** a Department of Pharmacology and Toxicology, College of Pharmacy, King Saud University, P.O. Box 22452, Riyadh, 11495, Saudi Arabia; b College of Pharmacy, King Saud University, P.O. Box 22452, Riyadh, 11495, Saudi Arabia; c Department of Anatomy, College of Medicine, King Saud University, P.O. Box 2925, Riyadh, 11461, Saudi Arabia

**Keywords:** Valproate, Indole-3-acetic acid, Chenodeoxycholic acid, Nrf2/HO-1, Cytoglobin

## Abstract

**Background:**

Purpose: Valproate (VPA) is an antiepileptic drug widely used to treat various psychiatric and neurological disorders. Although its use is generally considered safe, chronic administration may lead to kidney injury. The mechanisms underlying VPA kidney toxicity are not entirely explored. This has prompted our investigation into a novel molecular signaling pathway involved in VPA-induced kidney injury and the exploration of strategies to ameliorate this toxicity using indole-3-acetic acid (IAA) and chenodeoxycholic acid (CDCA).

**Methods:**

Rats were divided as follows: group I (control); group II (VPA group), where rats were administered VPA (500 mg/kg, i.p.) daily to induce kidney injury for 3 weeks; and groups III and IV, where rats were orally treated with either IAA (40 mg/kg) or CDCA (90 mg/kg), respectively, 1h post-VPA dose, for 3 weeks. The effects of these compounds on kidney tissues were evaluated with a focus on their antioxidant and anti-inflammatory properties using biochemical, histopathological, and immunohistochemical analyses.

**Results:**

VPA caused a significant reduction in renal glutathione (GSH) and heme oxygenase-1 (HO-1) levels, and superoxide dismutase (SOD) activity, along with a significant elevation in malondialdehyde (MDA) levels. Similarly, tumor necrosis factor-α (TNF-α), interleukin-1beta (IL-1β), and interleukin-6 (IL-6) levels were significantly increased. Immunohistochemical analysis demonstrated a significant decline in the immunoreactivity of nuclear factor erythroid 2-related factor (Nrf2) and cytoglobin antigens in renal cells. However, administration of either IAA or CDCA significantly ameliorated these altered parameters, including Nrf2/HO-1 and cytoglobin levels.

**Conclusion:**

IAA and CDCA alleviated the kidney injury induced by VPA via downregulating the inflammatory response and upregulating the antioxidant capacity in renal tissue.

## Graphical abstract

**Figure:**
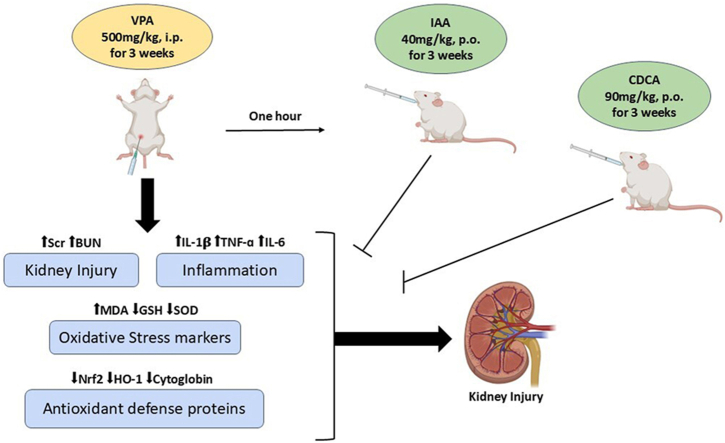

**Table:** List of Abbreviations:

Abbrev.	Definition	Abbrev.	Definition
VPA	Valproate	Nrf2	Nuclear factor erythroid 2-related factor
IAA	Indole-3-acetic acid	ROS	Reactive oxygen species
CDCA	Chenodeoxycholic acid	IACUC	Institutional Animal Care and Use Committee
GSH	Glutathione	CMC	Carboxymethylcellulose
SOD	Superoxide dismutase	CO_2_	Carbon dioxide
HO-1	Heme oxygenase-1	TBA	thiobarbituric acid
MDA	Malondialdehyde	TNB	5-thio-2-nitrobenzoic acid
TNF-α	Tumor necrosis factor-α	H_2_O_2_	Hydrogen peroxide
IL-1β	Interleukin-1 beta	BUN	Blood urea nitrogen
IL-6	Interleukin-6	FXR	Farnesoid X receptor

## 1 Introduction

Valproate (VPA) is an antiepileptic drug widely used to treat various psychiatric and neurological disorders. It is used in multiple therapeutic applications including certain types of seizures, bipolar disorder, and migraine prevention. VPA exerts its effects by potentiating GABA receptors and inhibiting voltage-gated sodium channels [1]. While generally considered safe, chronic administration may lead to adverse effects such as dermatologic reactions, bone marrow suppression, altered hepatic or pancreatic enzymes, and a potential risk of fetal malformation [2]. VPA induces tubular kidney injury by increasing reactive oxygen species (ROS), that mediate several signaling transduction cascades, activating transcription factors, and initiating apoptosis [3].

There is a link between VPA treatment and oxidative stress, as well as mitochondrial dysfunction [4]. However, the signaling cascade implicated in VPA-induced kidney injury has not been fully investigated. This prompted our interest in exploring novel molecular mechanisms involved in VPA-induced kidney injury and how to ameliorate it using indole-3-acetic acid (IAA) and chenodeoxycholic acid (CDCA), as these compounds have shown potential to provide both anti-inflammatory and antioxidant effects.

IAA, auxin, is the most common naturally occurring phytohormone that plays a role in cell growth and differentiation [5]. It is a transcriptional factor commonly expressed by immune cells that can regulate gut immune homeostasis [6], and prevent dysbiosis [7]. CDCA, a primary bile acid, serves as a signaling molecule in multiple physiological functions and possess anti-inflammatory activity [8,9].

There is a dearth of studies investigating the molecular mechanisms underlying the renoprotective activity of IAA or CDCA. Therefore, we hypothesized that IAA and CDCA could alleviate renal injury induced by VPA by modulating the Nrf2/HO-1 and cytoglobin pathways, which play critical roles in regulating cellular redox balance and inflammation-key mechanisms involved in VPA-induced kidney injury. Targeting these pathways holds immense promise of therapeutic benefits. This study is the first to examine the significance and implications of the Nrf2/HO-1 and cytoglobin pathways in the protective efficacy of IAA and CDCA against VPA-induced kidney injury.

## 2 Methods

### 2.1 Animals and experimental design

A total of 24 adult male Wistar rats with weights (160 ± 20 g) were obtained from the animal laboratory center at Pharmacy College, King Saud University. The rats were housed under standard temperature and humidity conditions with equal light and dark day cycles and had free access to food and water. The study was approved by the Research Ethics Committee (KSU‐SE‐20‐72), dated December 06, 2020.

Rats were allocated into four groups, 6 rats/each, as follows: **Group I** (Control); **Group II** (VPA): rats were injected VPA; **Group III** (VPA-IAA) and **Group IV** (VPA-CDCA): rats were treated orally with either IAA or CDCA 1 h post-VPA injection for 21 days. The control group received a vehicle 1 % carboxymethylcellulose (CMC). The other groups received i.p. injection of VPA at a dose of 500 mg/kg/day [10]; The VPA group received only VPA. The IAA-treated group received a daily oral dose of IAA (40 mg/kg) [11,12]. The CDCA-treated group was given a daily oral dose of CDCA (90 mg/kg) [13].

On day 22, the rats were euthanized by carbon dioxide (CO_2_) inhalation, and the whole trunk blood and kidneys were immediately collected. For serum separation, blood samples were allowed to coagulate at room temperature, and spun at 3000 rpm for 30 min in a precool centrifuge. The upper layers were carefully aspirated and stored at −80 °C for subsequent analyses. The collected kidney samples were divided into several parts, some were fixed in 10 % phosphate-buffered formaldehyde for histological and immunohistochemical (IHC) analyses, while other parts were homogenized in Tris–HCl buffer on ice, spun to remove tissue debris, and the supernatants were stored at −80 °C.

### 2.2 Kidney function analysis

Serum creatinine (Cat # 033 K-240) and blood urea nitrogen (Cat # 020–300) were evaluated in serum using colorimetric assay kits from United Diagnostics Industry Company.

### 2.3 Biochemical analysis of oxidative stress markers in kidney tissues

Malondialdehyde (MDA), is a main byproduct of the lipid peroxidation process during redox imbalance. Biochemical interaction between MDA that is present in the tissue and thiobarbituric acid (TBA) under acidic conditions and high temperature, forms a colored complex that can be quantified spectrophotometrically [14]. Glutathione (GSH) level was measured using Ellman's method, which is based on the reaction between 5,5′-dithiobis-(2-nitrobenzoic acid) or DTNB and sulfhydryl groups in GSH, resulting in bright yellow-colored solution measured spectrophotometrically at 412 nm [15]. The superoxide dismutase (SOD) activity assay is based on the ability of SOD to inhibit the autoxidation of pyrogallol, following the method described by Gao et al. [16]. SOD exerts its antioxidant effect by converting superoxide free radicals into less harmful hydrogen peroxide and oxygen. The presence of SOD reduces the rate of pyrogallol autoxidation by scavenging superoxide radicals, and the activity of the reaction was measured spectrophotometrically.

### 2.4 Immunological assay of tumor necrosis factor-α (TNF-α), interleukins (IL-6, IL-1β), and heme oxygenase-1 (HO-1) levels in kidney tissues

The expression levels of TNF-α (MBS590025), IL-6 (MBS2021530), and IL-1β (MBS2023030), and HO-1 (MBS764989) were assessed in kidney homogenate using highly sensitive enzyme-linked immunosorbent assay (ELISA) kits obtained from MyBioSource (San Diego, USA).

### 2.5 Histopathology and immunoreactivity analyses

The prefixed kidney samples were processed, embedded into the paraffin and then sectioned into 5-μm thickness on microscopic slides. Some sections were deparaffinized, rehydrated and subsequently stained with hematoxylin and eosin (H&E) to stain the nuclei and cytoplasm. The rest of the sections were subjected to preheated citrate buffer for epitope retrieval to examine changes in the immunoreactivity of cytoglobin (sc-365246) and Nrf2 (sc-518036) antibodies in the kidney. Endogenous peroxidase activity was quenched using 0.3 % hydrogen peroxide for half an hour. After blocking, the sections were kept with target antibodies overnight at 4 °C. The next day, the sections were rinsed thoroughly with a permeabilizing solution and kept with a biotinylated antibody (BA-2000, Vector Laboratories) for an hour at room temperature. The immunoreactive signal was amplified by ABC kit from Vector Laboratories. The peroxidase activity was visualized as a brown color precipitate using DAB Substrate. Finally, the slides were counterstained, dehydrated and mounted.

### 2.6 Statistical analysis

The results were presented as mean ± standard error of the mean (SEM) using Prism 9. Group comparison was carried out by one-way analysis of variance (ANOVA), followed by Tukey's test. Quantitative analyses of the histopathological and immunological examinations were performed using photographed pictures (×400 microscopic magnification). Differences were deemed statistically significant at P ≤ 0.05.

## 3 Results

### 3.1 Effect of IAA or CDCA on serum creatinine and BUN levels in VPA-induced kidney injury

**Fig. 1:**
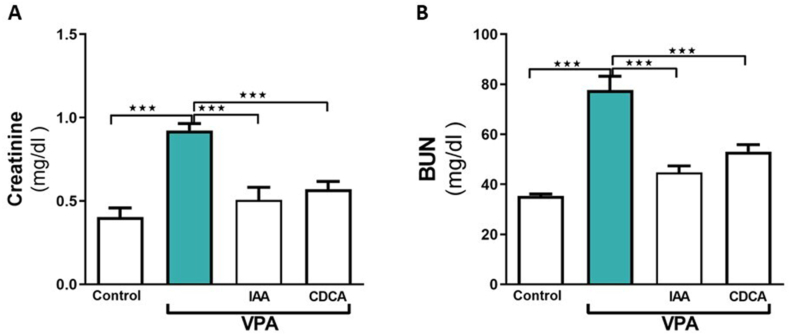
Effect of IAA or CDCA on the serum creatinine and BUN levels in VPA-induced kidney injury in rats. Data are expressed as mean ± SEM (n = 6). ∗∗∗P < 0.001.

Serum creatinine and BUN levels are established diagnostic indices for kidney injury. As depicted in Fig. 1 A and 1B, the levels of kidney markers were significantly elevated following VPA administration in comparison to the corresponding markers in the control group (∗∗∗P < 0.001). Treatment with IAA or CDCA following VPA significantly reduced serum creatinine and BUN levels compared with VPA alone (∗∗∗P < 0.001).

### 3.2 Effect of IAA or CDCA on kidney IL-6, IL-1β, and TNF-α levels in VPA-induced kidney injury

**Fig. 2:**
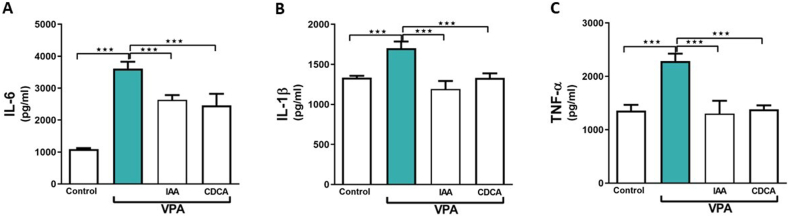
Effect of IAA or CDCA on kidney IL-6, IL-1β, and TNF-α levels in VPA-induced kidney injury in rats. Data are expressed as mean ± SEM (n = 6). ∗∗∗P < 0.001.

As presented in Fig. 2 A, B, and C, the administration of VPA significantly increased the renal tissue levels of IL-6, IL-1β, and TNF-α relative to the control rats (∗∗∗P < 0.001). Treating rats with IAA or CDCA significantly reduced the renal levels of the aforementioned inflammatory indices relative to VPA-intoxicated rats (∗∗∗P < 0.001).

### 3.3 Effect of IAA or CDCA on kidney MDA, GSH, HO-1 levels, and SOD activity in VPA-induced kidney injury

**Fig. 3:**
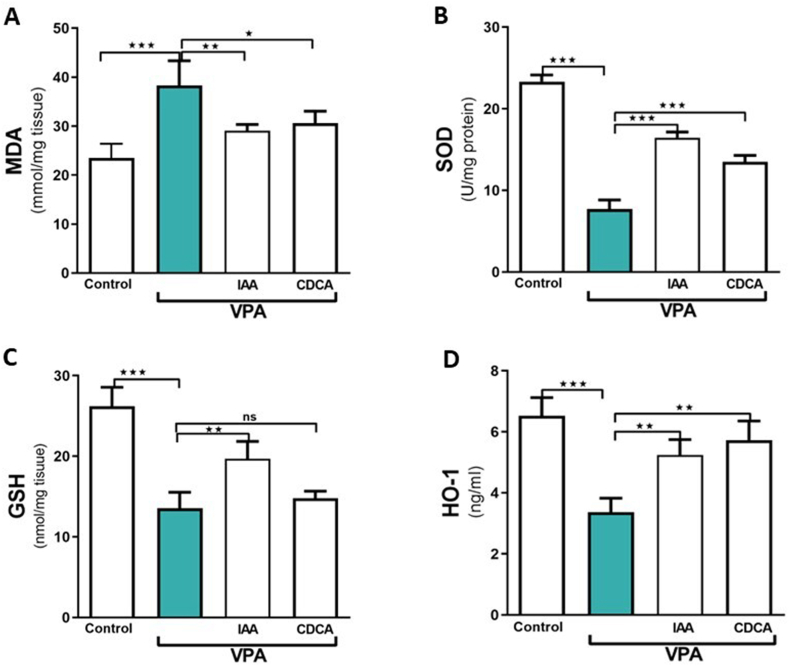
Effect of IAA or CDCA on the kidney levels of MDA, SOD, GSH, and HO-1 in VPA-induced kidney injury in rats. Data are expressed as mean ± SEM (n = 6). ∗∗∗P < 0.001, ∗∗P < 0.01 and ∗P < 0.05. ns; no significance.

VPA administration significantly increased renal MDA levels compared to the control group (∗∗∗P < 0.001), whereas concurrent administration of IAA or CDCA with VPA overdose showed a significant reduction in MDA levels compared with the VPA-administered group (∗∗P < 0.01, and ∗P < 0.05, respectively (Fig. 3 A)). Additionally, the renal levels of GSH, HO-1, and SOD activity were considerably decreased following VPA treatment (∗∗∗P < 0.001), whereas treatment with IAA remarkably restored SOD activity (∗∗∗P < 0.001) and GSH (∗∗P < 0.01) and HO-1 (∗∗P < 0.01) levels (Fig. 3 B, C, and D). Treatment with CDCA produced a significant elevation in the levels of HO-1 (∗∗P < 0.01) and SOD activity (∗∗∗P < 0.001); however, it failed to restore the GSH level relative to the VPA-administered group (Fig. 3B, C, and D).

### 3.4 IAA or CDCA mitigates the renal histopathological alteration accompanied by VPA overdose

**Fig. 4:**
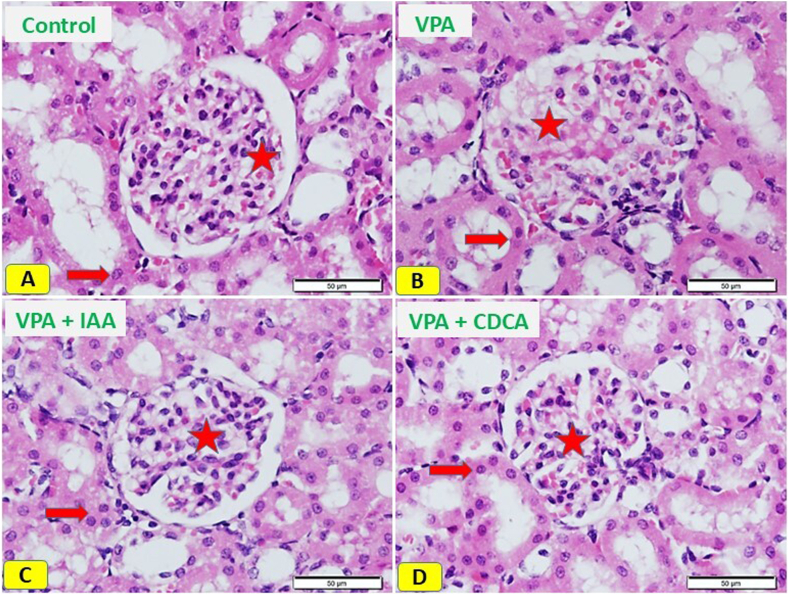
IAA or CDCA mitigates the morphological changes in VPA-induced kidney injury. Photomicrographs of H&E-stained kidney sections. (A) Control rats showing normal cells of renal corpuscle (star), and tubular cells (arrow). (B) Sections from rats exposed to VPA showing marked degeneration of many cells of the glomerulus of renal corpuscle (star), and renal tubular epithelium (arrow). (C) Kidney sections from rats exposed to VPA and IAA, showing regeneration of glomerulus epithelium of the renal corpuscle (star), and tubular epithelium (arrow). (D) Kidney sections from rats exposed to VPA and treated with CDCA showing apparently normal cells of the glomerulus (star) and tubular wall epithelium cells (arrow) (Scale bar; 50 μm).

H&E-stained kidney sections of normal control rats exhibited normal renal corpuscles and tubular cells, as presented in Fig. 4 A. In contrast, kidney sections from rats administered VPA exhibited marked degeneration of many cells in the glomerulus of the renal corpuscle and renal tubular epithelium (Fig. 4 B). Kidney sections from rats administered VPA and treated with IAA exhibited apparent improvements in renal tissue, with normal cells in the glomerular and tubular wall epithelium (Fig. 4 C). Lastly, rats administered VPA and treated with CDCA demonstrated regeneration of the glomerular epithelium in the renal corpuscle, as well as the tubular epithelium (Fig. 4 D). Quantitative analysis of kidney sections revealed a marked increase in degenerated renal cells after VPA exposure, whereas the groups received either IAA or CDCA revealed less number of degenerated cells (Table 1).

**Table 1:** Quantification of degenerated epithelial cells stained with H&E.

Groups	Quantification of Degenerated cells
**Control**	**5 ± 0.63**
**VPA**	**49.2 ± 0.94** ∗∗∗∗
**VPA + IAA**	**11.5 ± 0.71**^**π**^^**π**^^**π**^^**π**^
**VPA + CDCA**	**20.6 ± 0.79**^**π**^^**π**^^**π**^^**π**^

Data are expressed as mean ± SEM, ∗∗∗∗P < 0.001 compared to control group, and ^**π**^^**π**^^**π**^^**π**^ P < 0.001 compared to VPA group.

### 3.5 Effect of IAA or CDCA on the cytoglobin expression in VPA-induced kidney injury in rats

**Fig. 5:**
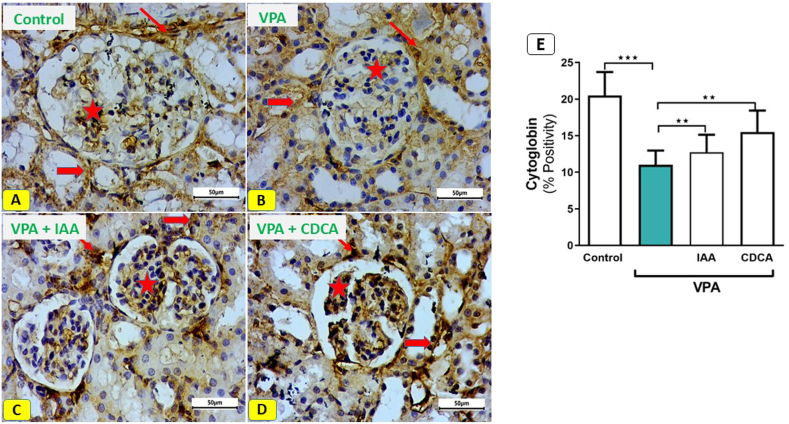
Effect of IAA or CDCA on cytoglobin expression in VPA-induced kidney injury in rats. Photomicrographs of cytoglobin immune stained kidney sections. (Scale bar; 50 μm). (A) control rats showing a normal immune reaction of the cells of the renal corpuscle (star), interstitial tissue (thin arrow) and cytoplasm of tubular cells (thick arrow). (B) sections from rats exposed to VPA showing a marked decrease of the immune reactivity in cells of renal corpuscle (star), interstitial tissue (thin arrow) and renal tubule cytoplasm (thick arrow). (C) kidney sections from rats exposed to VPA and IAA, showing recovery of the immune reactivity in the renal corpuscle (star), tubular cytoplasm (thick arrow) and interstitial tissue (thin arrow). (D) kidney sections from rats exposed to VPA and CDCA showed an apparent recovery of the immune reactivity in the renal corpuscle (star), tubular cytoplasm (thick arrow) and interstitial tissue (thin arrow). (E) a quantitative analysis revealed a significant increase in the cytoglobin expression in the groups treated with either IAA or CDCA compred to VPA-intoxicated group.

Control kidney sections showed normal cytoglobin expression in the renal corpuscle, interstitial tissue, and tubular cells, with a positive immune response (Fig. 5 A). In contrast, kidney sections from VPA-administered rats exhibited a marked decrease in immune reactivity in the cells of the renal corpuscle, interstitial tissue, and renal tubular cytoplasm. (Fig. 5 B). Kidneys from VPA + IAA-treated rats showed improved immune reactivity in the renal corpuscle, tubular cytoplasm, and interstitial tissue (Fig. 5 C). Similarly, kidneys from VPA + CDCA rats exhibited recovery of normal immune reactivity in these regions (Fig. 5 D). Quantitative analysis revealed a significant increase in cytoglobin expression in kidney sections obtained from rats that received either treatment compared to VPA-administered rats (Fig. 5 E).

### 3.6 Effect of IAA or CDCA on the renal Nrf2 expression after VPA-induced kidney injury in rats

**Fig. 6:**
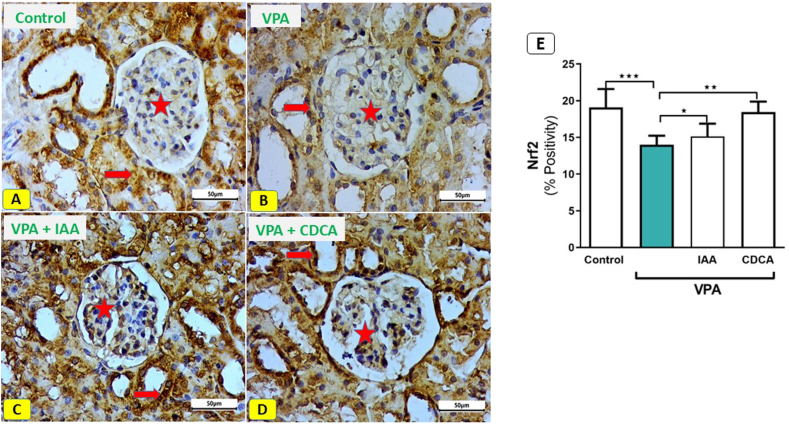
Effect of IAA or CDCA on Nrf2 expression in VPA-induced kidney injury in rats. Photomicrographs of Nrf2 immune stained kidney sections. (Scale bar; 50 μm). (A) Control rats showing normal immune reaction of the cells of renal corpuscle (star), interstitial tissue and cytoplasm of tubular cells (thick arrow). (B) Sections from rats exposed to VPA showing a marked decrease of the immune reactivity in cells of renal corpuscle (star), interstitial tissue, and renal tubule cytoplasm (thick arrow). (C) Kidney sections from rats exposed to VPA and IAA showed a recovery of the immune reactivity in the renal corpuscle (star), and tubular cytoplasm (thick arrow) and, interstitial tissue. (D) Kidney sections from rats exposed to VPA and CDCA revealed a recovery of the immune reactivity in the renal corpuscle (star) and tubular cytoplasm (thick arrow), and interstitial tissue. (E) a quantitative analysis revealed a significant increase in the Nrf2 expression in the rats treated with either IAA or CDCA compred to VPA-intoxicated rats.

In control rats, Nrf2-immunostained kidneys revealed normal Nrf2 expression in the renal corpuscle, interstitial tissue, and tubular cytoplasm, with a positive immune response (Fig. 6 A). In contrast, kidney sections from VPA-administered rats exhibited a marked decrease in immune reactivity within the renal corpuscle, interstitial tissue, and renal tubular cytoplasm. (Fig. 6 B). The use of IAA following VPA exposure improved Nrf2 immune reactivity in the renal corpuscle, tubular cytoplasm, and interstitial tissue (Fig. 6 C). Additionally, kidney sections from VPA + CDCA rats demonstrated recovery of typical immune reactivity in the renal corpuscle, tubular cytoplasm, and interstitial tissue (Fig. 6 D). As shown in Fig. 6 E, the protein expression of Nrf2 in the kidney was significantly increased in rats that received either IAA or CDCA in comparison to the VPA-intoxicated rats.

## 4 Discussion

VPA has been used for decades as an antiepileptic agent. Despite its excellent efficacy in treating psychiatric disorders, many adverse effects have been associated with its chronic use [17]. Limited information is available regarding the mechanisms underlying VPA-induced kidney damage. This gap in knowledge prompted us in investigating the signaling pathways involved and exploring renoprotective strategies to mitigate the risk of VPA-induced kidney injury. Given the established roles of oxidative stress and inflammation in kidney injury, we hypothesized that IAA and CDCA might potentially alleviate VPA-induced kidney injury by targeting theses pathways.

Serum creatinine and BUN are classical biomarkers used to assess renal function and detect renal injury [18]. In this study, notable elevations in serum creatinine and BUN levels were observed following VPA administration, confirming its nephrotoxic effects. These findings are consistent with those of Heidari et al., who reported an increase in both markers following VPA treatment in rats [19]. The administration of IAA and CDCA markedly decreased the serum levels of creatinine and BUN, indicating their early protective roles against VPA. The effects of IAA on kidney function remain controversial. A study by Ismail [20] demonstrated increased urea and creatinine levels at a high dose of IAA, which is more than 12 times the dose typically used in our study. However, Satoh et al., suggested that IAA may exacerbate existing kidney damage [21]. Currently, there is no research on the effects of low-dose IAA on urea and creatinine levels. Remarkably Cernaro et al. found that IAA increases cell proliferation in LLC-PK1 cell line, suggesting a regenerative activity of IAA [22]. Similarly, CDCA has been found to possess nephroprotective effects, as demonstrated by its ability to reverse cisplatin-induced elevations of both kidney markers [23]. Histopathological examination of VPA-treated renal tissue was performed to further verify the potential renoprotective roles of IAA and CDCA. Consistent with the renal biomarker results, both IAA and CDCA promoted regeneration of damaged renal corpuscles and tubular cells following VPA exposure, restoring tissue architecture.

To further investigate the renoprotective mechanisms of IAA and CDCA, inflammatory markers were assessed. In this study, the expression of inflammatory cytokines significantly increased following VPA administration. Emerging evidence has demonstrated the contribution of inflammation to VPA-induced injury [24]. Our findings align with previous reports showing that VPA elevates the production of inflammatory cytokine, including IL-6 [25]. In contrast, a low dose of VPA has demonstrated nephroprotective effects in mice with renal injury [26]. This may be attributed to differences in the dosage and treatment duration. Treatment with either IAA or CDCA attenuated the inflammatory response by downregulating the expression of IL-6, IL-1β, and TNF-α, suggesting that their renoprotective effects are mediated through interference with inflammatory pathways. This result is consistent with those of earlier studies confirming anti-inflammatory effect of IAA in a collagen-induced arthritis mouse model [27]. In an ankylosing spondylitis mouse model, IAA reduced inflammation by suppressing TNF-α, IL-6, IL-17A, and IL-23, while enhancing the expression of IL-10 [7]. Similarly, CDCA demonstrated anti-inflammatory effects by inhibiting the expression of TNF-α and IL-6 in renal-injured rats [28].

Regarding oxidative stress, the current study revealed an upregulation of oxidative biomarkers in rats treated with VPA, characterized by increased MDA levels and decreased GSH and SOD levels in kidney tissues. A previous study by Chaudhary et al. reported that VPA-induced renal tissue injury is associated with increased lipid peroxidation and reduced GSH levels [29]. The oxidative imbalance detected following VPA treatment was reversed by both IAA and CDCA, IAA showing a more pronounced effect. Additionally, antioxidant markers, including GSH and SOD, were improved following IAA treatment, suggesting its potential to restore normal oxidative state in VPA-treated rats. Our recent study demonstrated the antioxidant activity of both IAA and CDCA in hepatic tissues by restoring the levels of GSH and SOD [11]. Consistent with this, IAA has been reported to mitigate hepatic oxidative imbalances and improve the antioxidant capacity in rats through similar mechanisms [30]. The renoprotective role of IAA in VPA-treated rats may be attributed to its free radical-scavenging capacity [31].

To further explore the novel molecular mechanisms involved in VPA-induced kidney injury, we investigated the implications of the Nrf2/HO-1 and cytoglobin signaling pathways. Growing evidence has demonstrated the roles of Nrf2/HO-1 and cytoglobin signaling in oxidative stress [32]. The cellular antioxidant system is primarily regulated by Nrf2, which modulates the expression of cytoprotective genes, including HO-1, to reduce the production of ROS and overall oxidative stress [33]. Therefore, identifying agents that target the Nrf2/HO-1 pathway may provide a protective strategy against oxidant initiator, such as VPA. Remarkably, the results of our study revealed that IAA and CDCA upregulated the expression of the Nrf2/HO-1 signaling pathway, contributing to their renoprotective effects. Similarly, IAA protects against H_2_O_2_-induced toxicity in human dental pulp stem cells through overexpressing Nrf2/HO-1 signaling [31]. Notably, the modulation of Nrf2/HO-1 signaling by CDCA in kidney cells is a novel discovery that offers a promising approach toward renoprotection against VPA toxicity. In our study, the effect of CDCA was superior to that of IAA in reversing the VPA-induced oxidative imbalance of HO-1. A possible explanation may be the ability of CDCA as a farnesoid X receptor (FXR) agonist, to directly activate Nrf2, which in turn regulates the expression of antioxidant enzymes, including HO-1 [34]. Furthermore, the current investigation found that both IAA and CDCA reversed VPA-induced downregulation of cytoglobin. This is the first study to confirm the implication of cytoglobin expression in VPA-induced kidney injury and further explain the molecular mechanism by which IAA and CDCA exert their protective effects in kidney tissue. Cytoglobin, a member of globin family, is a redox-sensitive protein found in several body tissues, including the kidneys [35]. It exhibits high SOD activity and maintains intracellular oxygen levels through its ROS-detoxifying properties [36]. The downregulation of cytoglobin has been shown to exacerbate disease states [37]. Additionally, previous research has demonstrated that umbelliferone, a phytochemical compound, exerts its nephroprotective effect against cisplatin-induced kidney injury through various mechanisms, including cytoglobin upregulation [33]. Therefore, cytoglobin may serve as a useful therapeutic target against VPA-induced injury.

## 5 Conclusion

Collectively, the results of this study provide an *in vivo* evidence supporting the renoprotective roles of IAA and CDCA against VPA overdose. Concurrent supplementation of either IAA or CDCA during VPA treatment might potentially improve safety profile of VPA by mitigating the adverse renal effects associated with its use. IAA and CDCA exert protective effects through several pathways, including the modulation of oxidative stress and inflammatory responses, and the expression of Nrf2/HO-1, and cytoglobin.

One major limitations of animal research is the sample size. In our study, we calculated the total number of animals required and successfully used an ethically acceptable sample size to achieve the scientific objectives.

## CRediT authorship contribution statement

**Ahlam M. Alhusaini:** Writing – review & editing, Writing – original draft, Validation, Supervision, Project administration, Methodology, Funding acquisition, Conceptualization. **Wedad Sarawi:** Writing – review & editing, Writing – original draft, Validation, Project administration, Methodology, Formal analysis. **Noor Mukhtar:** Writing – review & editing, Writing – original draft, Methodology. **Danah Aljubeiri:** Writing – review & editing, Writing – original draft, Methodology. **Amjad S. Aljarboa:** Writing – review & editing, Writing – original draft, Methodology. **Hessa Alduhailan:** Writing – review & editing, Writing – original draft. **Faris Almutairi:** Writing – review & editing, Writing – original draft, Validation. **Raeesa Mohammad:** Writing – review & editing, Methodology, Formal analysis. **Muhammad Atteya:** Writing – review & editing, Methodology, Formal analysis. **Iman Hasan:** Writing – review & editing, Writing – original draft, Validation, Methodology, Formal analysis.

## Data availability statement

Data generated during this study are available upon request from the corresponding author.

## Declaration of competing interest

The authors declare that they have no known competing financial interests or personal relationships that could have appeared to influence the work reported in this paper.
